# Social Priming Increases Nonverbal Expressive Behaviors in Schizophrenia

**DOI:** 10.1371/journal.pone.0109139

**Published:** 2014-10-02

**Authors:** Jonathan Del-Monte, Stéphane Raffard, Delphine Capdevielle, Robin N. Salesse, Richard C. Schmidt, Manuel Varlet, Benoît G. Bardy, Jean-Philippe Boulenger, Marie-Christine Gély-Nargeot, Ludovic Marin

**Affiliations:** 1 Movement to Health Laboratory, EuroMov, Montpellier-1 University, Montpellier, France; 2 University Department of Adult Psychiatry, Hôpital de la Colombière, CHU Montpellier, Montpellier-1 University, Montpellier, France; 3 Epsylon, Laboratory Dynamic of Human Abilities & Health Behaviors, Department of Sport Sciences, Psychology and Medicine, University of Montpellier, Montpellier, France; 4 Department of Psychology, College of the Holy Cross, Worcester, MA, United States of America; 5 INSERM U-1061, Montpellier, France; 6 Institut Universitaire de France, Paris, France; 7 The MARCS Institute, University of Western Sydney, Sydney, Australia; Benito Menni Complejo Asistencial en Salud Mental, Spain

## Abstract

Semantic priming tasks are classically used to influence and implicitly promote target behaviors. Recently, several studies have demonstrated that prosocial semantic priming modulated feelings of social affiliation. The main aim of this study was to determine whether inducing feelings of social affiliation using priming tasks could modulate nonverbal social behaviors in schizophrenia. We used the Scrambled Sentence Task to prime schizophrenia patients according to three priming group conditions: pro-social, non-social or anti-social. Forty-five schizophrenia patients, diagnosed according to DSM-IV-TR, were randomly assigned to one of the three priming groups of 15 participants. We evaluated nonverbal social behaviors using the Motor-Affective subscale of the Motor-Affective-Social-Scale. Results showed that schizophrenia patients with pro-social priming had significantly more nonverbal behaviors than schizophrenia patients with anti-social and non-social priming conditions. Schizophrenia patient behaviors are affected by social priming. Our results have several clinical implications for the rehabilitation of social skills impairments frequently encountered among individuals with schizophrenia.

## Introduction

Semantic priming is an experimental procedure in which exposure to a stimulus influences a response to a later stimulus. Semantic priming has been the standard paradigm for evaluating the associations among concepts stored in semantic memory [1]. A ‘priming effect’ classically refers to a change in reaction time to answer a stimulus word after having been exposed to a previous related word (i.e. Cat-Dog versus Cat-Leg). In the recent years, a great amount of evidence has been accumulated to suggest that priming tasks can be used to induce a feeling of social affiliation and to facilitate cooperative behaviors between two individuals [2]. For example, Lakin and Chartrand [3] showed that prosocial primes elicit stronger unconscious mimicry during social interaction. Young children were found to help a person in need more often, and more spontaneously, when primed with photographs evoking affiliation than when primed with photographs evoking individuality [4]. These results suggest that promoting social behaviors by activating social goal representations (e.g. nonverbal behaviors) may have interesting applications in patients with severe interpersonal impairments. Social deficits are a feature of many mental disorders, such as schizophrenia [5], characterized by a link between social functioning deficits and nonverbal expressiveness (spontaneous hand gestures and spontaneous smiles) [6]. Classically, Social deficits in schizophrenia have to date been treated by means of cognitive remediation or social skills training therapy. The former of these interventions has shown limited benefits [7]–[9] and, while the latter has shown effects on social cognition, relevant studies have been few [10], [11] and to our knowledge none of them have targeted nonverbal social behaviors.

Past studies investigating priming schizophrenia patients have shown conflicting results. In reaction time task toward a word target using a priming task, Rossell and David (2006) found a significant decrease in reaction time compared to healthy controls (hyperpriming) but some years before, the same authors found a significant increase in reaction time (hypopriming) [12]. Finally, other studies found a comparable reaction time in schizophrenia patients and healthy controls. These studies taken together point out that schizophrenia patient were as sensitive to priming effects as healthy individuals [13]. However, such studies only used the priming task to examine abnormal semantic network activation in schizophrenia and not as a therapeutic tool in this pathology.

Nevertheless, reductions in social motor behaviors play a role in social deficits in this pathology [6]. Nonverbal behavior impairments are often characterized by a reduction in the number of spontaneous gestures [14]–[17]. In particular, a recent review of Lavelle et al. (2014) showed that schizophrenia patients displayed fewer hand gestures and less variability and flexible facial expressions during social interactions. Additionally, nonverbal behavior reductions, in schizophrenia, were linked with a decrease in social competences [18] and social functioning [6]. Given the social deficits observed in this disorder, it seems essential to investigate the effect of social priming on nonverbal behaviors in schizophrenia.

The main aim of the current study was to determine whether social priming could modulate nonverbal social behaviors of patients suffering from schizophrenia. We evaluated nonverbal social behaviors using the Motor-Affective subscale of the Motor-Affective-Social-Scale (MASS) [19] in order to compare the effect of three kinds of social priming (pro-social, non-social and anti-social) on schizophrenia patients. We expected that in the pro-social priming condition, the number of nonverbal social behaviours in schizophrenia participants would be significantly increased compared to the non-social and the anti-social social priming conditions.

## Materials and Methods

### Participants

Forty-five schizophrenia patients diagnosed according to DSM-IV-TR criteria participated in this study. Diagnoses were established using the Structured Clinical Interview for DSM-IV (SCID-CV) [20]. Patients were in the stable phase of the illness according to the current treating psychiatrist, and categorized, as had no hospitalizations or changes in housing in the month prior to entering the study. All individuals with schizophrenia were receiving antipsychotic medication at the time of participation and were recruited from the University Department of Adult Psychiatry in Montpellier. Exclusion criteria for both groups were: (a) known neurological disease, (b) developmental disability, or (c) substance abuse in the past month. All participants were proficient in the French language, had normal or corrected-to-normal vision and naïve as to the purpose of the study. Participants gave written consent to participate in this experiment. The capacity of the patients to provide informed consent was established through a structured interview, and was also confirmed by their treating psychiatrists. The local Ethics Committee approved the study (CPP Sud Méditérannée III, Montpellier, France AFSSAPS 2009-A00513-54 24, 07/22/2009) conforming to the Declaration of Helsinki.

One day before testing, neurologic soft signs and extrapyramidal symptoms were rated using the Neurological Soft Signs Scale (NSS) [21], the level of depressive symptoms was assessed with the Beck Depression Inventory – II (BDI-II) [22], the premorbid IQ was evaluated using the French National Adult Reading Test (*f*NART) [23], and an indication of their social anxiety was obtained using the Liebowitz-Social-Anxiety-Scale (LSAS) [24]. Finally for schizophrenia patients, the severity of symptoms was assessed using the Positive And Negative Syndrome Scale (PANSS) [25] and social functioning was assessed by the modified prosocial subscale of the PANSS [26] (see Table 1 for participants' characteristics).

**Table 1:** Means ± standard deviation of demographic and clinical characteristics of participants.

	SZ patients (n = 15) Pro-social priming	SZ patients (n = 15) Anti-social priming	SZ patients (n = 15) Non-social priming	Statistics	*p*
Age (years)	35.33±11.30	35.66±11.10	32.06±8.37	*H* = 1.384	0.500
Sex ratio (M/F)	9/6	10/5	10/5	*X^2^* = 0.194	0.908
Education level (years)	12±3	12.53±2.64	11.20±3.16	*H* = 1.804	0.406
IQ premorbid (*f*NART)	105.80±8	107±7.60	107.26±10.29	*H* = 0.413	0.813
Depression level (BDI-II)	16.13±7.46	13.53±4.85	14.73±11.75	*H* = 1.172	0.557
Anxiety level (LSAS)	27.40±9.34	25.93±8.52	27.60±18.60	*H* = 0.035	0.983
Neurologic Soft Signs scores	9.26±4.78	12.10±6.04	12.63±5.26	*H* = 4.039	0.133
PANSS					
• Total	71.06±14.30	70.80±13.15	72.60±11.31	*H* = 0.207	0.902
• Positive	16.53±4.34	16.66±6.86	18.40±7.60	*H* = 0.649	0.723
• Negative	17.06±5.86	18.33±6.55	18.13±3.83	*H* = 1.068	0.586
• Psychopathology	37.46±7.67	35.80±6.12	36.01±4.43	*H* = 0.131	0.937
• Prosocial PANSS items	10±3.98	11.53±4.82	11.20±3.29	*H* = 0.842	0.656

SZ: Schizophrenia, IQ: Intellectual Quotient, *f*NART: French version of the National Adult Reading Test, BDI-II: Beck Depression Inventory version-II, LSAS: Liebowitz-Social-Anxiety-Scale, PANSS: Positive and Negative Syndrome Scale.

*H*: Kruskal-Wallis non-parametric test, *X^2^*: Chi-square parametric test.

### Procedure

Schizophrenia patients who were blind to the real goal of the experiment were randomly assigned to one of three conditions (pro-social, anti-social and non-social). Upon arrival at the laboratory, the participant had to complete one first task. The task was purportedly designed to examine the effect of color on the ability to form comprehensible sentences (priming task). A first experimenter, always the same for all patients and blind to the purpose of the study, verbally explained the instructions for each task. Participants were told that they should use four of the five words to make a complete sentence. They would have to say the sentence verbally and a new screen would appear with five different words in different colors.

Immediately after having completed the priming task (either the pro-social, anti-social or non-social condition), schizophrenia patients were tested for nonverbal social behaviors using the Motor-Affective subscale of the MASS by a second experimenter (always the same) blind to the specific conditions of priming to prevent any “un-blind experimenter effect” [27], [28].

### Material

#### Social priming task: Scrambled-sentence Task

**Figure 1:**
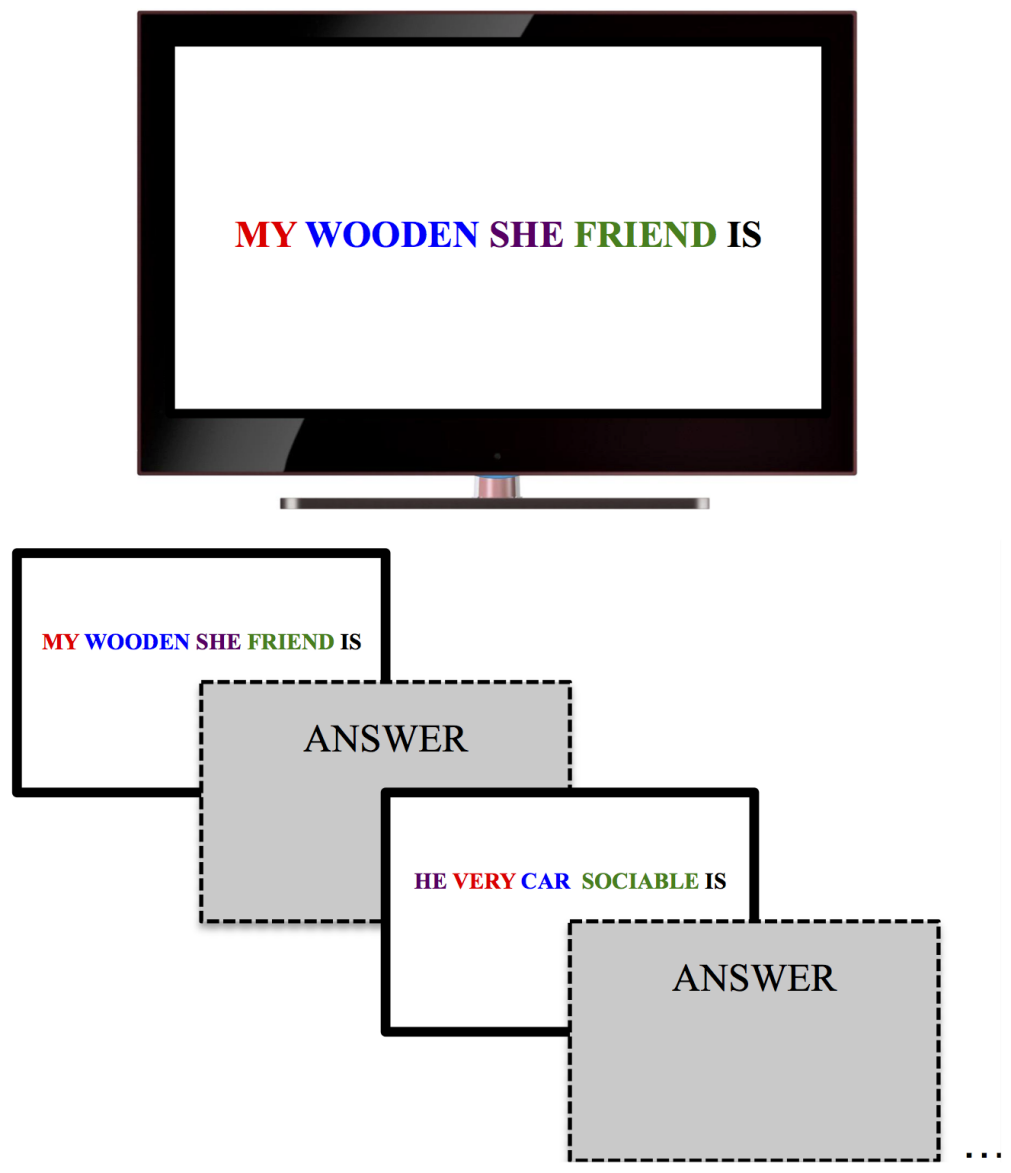
Experimental setup for the priming task. Participants sat on a chair in front of a screen. Participants were told that they should use four of the five words to make a complete sentence. They then would say the sentence verbally and a new screen would appear with five different words on it for the next trial.

Stimuli were presented on a computer screen (60 Hz,.45 m, 1280×800 pixels). Viewing was unrestrained at a distance of approximately.6 m. In the priming task, words were presented in color on a white background. Each trial showed five words arranged horizontally across the screen. Words were presented in Arial, font size 24 and the color of the words varied randomly over trials. They were presented in either green, blue, red, yellow or black (Figure 1). Three versions of the Scrambled-Sentence Task were constructed (see [29] for a similar procedure): One was intended to prime the pro-social attitude (affiliation), another the anti-social attitude (non-affiliation) and a third was intended to prime no attitude, the non-social priming condition. For all versions, 24 trials contained an adjective or a verb semantically related to the trait in question. For the pro-social version, the critical priming words were: *friendly, pleasant, states, altruist, friendship, friend, courteous, company, sociable, community, communicative, reunited, cooperative, dating, gathered, group, together, popularity, team, share, family, frequented, married, interaction*. For the anti-social version, the critical priming words were: *unique, individual, autonomous, lonesome, oneself, alone, separated, contained, disagreeable, seclusion, detached, uncooperative, divided, rebel, egocentric, selfish, personal, single, neglecting, unpopular, isolated, independent, locked, individually*. The non-social version consisted of the 24 non-social words. The non-social words were: *tomato, stone, box, branch, cloud, river, plank, light bulb, carpet, bag, button, fog, table, tree, wood, hill, spoon, radio, step, curtains, picture, wind, road, brick*.

Prior to the experiment, a pre-test was carried out to verify the semantic relationship between the target words and the attitudes that we intended to prime. Two separate samples, not included in the main experiment, of 10 schizophrenia patients and 10 healthy controls were asked to rate each word in terms of how social they considered it to be on a 7-point scale (-3 = *very anti-social*, 3 = *very pro-social*). A Friedman test indicated significant differences between pro-social, non-social and anti-social words in their perceived relation to sociality (*p*<.001). Results confirmed that the schizophrenia group and the healthy control group were equivalent in their judgments (pro-social words: *p* = .288; non-social words: *p* = .676 and anti-social words: *p* = .520) and that the stimulus words were strongly related to the attitudes that they were intended to prime.

#### Nonverbal social behavior task

Trémeau et al. (2008) developed the Motor-Affective-Social-Scale (MASS) for schizophrenia. The first part of this scale, the Motor Affective subscale of the MASS, assesses fundamental expressive deficits by evaluating spontaneous smiles, hand gestures, voluntary smiles and speech flow. The second part of the MASS assesses the lack of initiation (avolition) through motor retardation, personal hygiene, attendance at groups and verbal interaction. In our study, we used the first part of this scale to assess the social priming effect on nonverbal social behaviors in schizophrenia. An interviewer/rater (always the same), blind to the conditions of priming, assessed nonverbal social behaviors.

During a structured interview, participants were asked three questions in the same order. First, participants were asked to report everything they did the day before (for 2 min). Second, they were asked to describe a movie from memory (for 1 min). Third, they were asked to imagine what they would do if given one million Euros (for 30 s). After the interview, participants were asked to make their “most beautiful smile”, and pre-defined behavioral anchors were used to rate the posed smiling. Interviewers were instructed to ask the predefined questions each time the participant's speech flow stopped for 3 s. Four behaviors were recorded and rated: spontaneous smiles, spontaneous hand nonverbal gestures, speech flow and voluntary facial expression.

Smiles are defined as bilateral upper movements of both lip corners (contraction of the zygomatic muscle) with or without contraction of the orbicularis oculi muscle. The rules to rate one smile were i) count one smile if you observe a clear smiling expression or an expression that gives you an impression of smiling (it is not always possible for the interviewer to identify the muscular movement accompanying a smiling expression during an interview), ii) do not consider intensity and iii) do not consider the communicative value of smiles (even a smile that appears to be sarcastic counts as one, if both lip corners move). The rules to count two distinct smiles were i) two smiles should be differentiated by a resting period of two seconds and ii) if smiling is continuous, count one new smile every three seconds.

Spontaneous hand gestures are defined as hand movements directly tied to speech to illustrate or stress what is being said (also called “illustrators”) or to replace a word or a sentence, such as pointing with a finger or waving a hand to mean “no” (also called “emblems”). Nonverbal gestures should be differentiated from adaptors (non-communicative, self-stimulating, hand-to-body gestures, such as scratching, touching face with hand, hair playing, hand rubbing, playing with buttons) and from abnormal movements (tremors, dyskinesia, and dystonia). Rules to rate one nonverbal gesture were i) one gesture can involve hand movement only (at the wrist level) or arm movement with or without wrist movements. For movements of fingers only, rate emblematic movements only (such as movements of fingers to say “no”, or to express numbers), ii) hand nonverbal gestures must be of enough amplitude to be clearly seen: small movements of wrists that are hardly visible should not be counted and iii) rate occurrences of unilateral or bilateral hand gestures indistinctively (bilateral hand movements that occur at the same time only count for one occurrence). Rules to count two distinct hand nonverbal gestures were i) if a resting period (hands on chair or body, or hands and arms kept in the same position for at least two seconds) is observed between two gestures, ii) if the same gesture continues for more than three seconds, count one gesture every three seconds and iii) if two gestures are tied to two different ideas or words: for example hand nonverbal gestures accompanying the following speech: “I went there, and then came back here” count as two gestures.

The number of questions or instructions coming from the interviewer assessed speech flow. Rules to count one question were i) do not rate interviewer's first instruction, questions or instructions irrelevant to the conversation theme (such as: “Could you speak louder?” “Could you take your hands out of your pockets?”), and comments (such as “You are doing well”), ii) if the subject shows looseness of associations or tangentiality, or does not answer your question, you should repeat the same question once (do not count it) and if necessary ask a different question (counts as a new question) and iii) if the subject's speech is very slow with pauses more than 5 seconds long between words or sentences, the interviewer should express back-channel verbal communication such as “and?”, “yes?”, “OK”, “go ahead” and must count it as one question (this does not apply to the subject's first sentence, just after interviewer's instruction). Rules to count two distinct questions were i) the same question or instruction asked on two separate occasions during the interview counts twice; for example “Tell me more about this”, “What else did you do this morning?”.

Voluntary smile was defined as the voluntary action of smiling. The facial expression must give the impression of a smile (grimaces even involving the contraction of the orbicularis oculi muscle should not be counted as a smile). A small smile can be defined as a smile in which each lip corner does not move up more than 2 mm from resting position. The contraction of the orbicularis oculi muscle leads to the bulging of the inferior eyelid and to the squinting of the eye (Crow's feet). If the subject has wrinkles at the corner of his/her eyes, look for an increase in the eyes squinting.

Each occurrence of these behaviors was transferred on a Likert scale of 1–4. Total number of spontaneous smiles was rated as follows: i) 0 smile corresponded to a score of 1 on the Likert scale, ii) 1–2 smiles corresponded to a score of 2 on the Likert scale, iii) 3–5 smiles corresponded to a score of 3 on the Likert scale and iiii) 5 smiles and above corresponded to a score of 4 on the Likert scale. Total number of spontaneous hand nonverbal gestures was rated as follows: i) 0–3 hand gestures corresponded to a score of 1 on the Likert scale, ii) 4–9 hand gestures corresponded to a score of 2 on the Likert scale, iii) 10–20 hand gestures corresponded to a score of 3 on the Likert scale and iiii) 21 hand gestures and above corresponded to a score of 4 on the Likert scale. Total number of questions was rated as follows: i) 0–1 question corresponded to a score of 4 on the Likert scale, ii) 2–4 questions corresponded to a score of 2 on the Likert scale, iii) 5–7 questions corresponded to a score of 3 on the Likert scale and iiii) 8 questions and above corresponded to a score of 1 on the Likert scale. The quality of voluntary smile was rated as follows: i) « no expression OR smile only » corresponded to a score of 1 on the Likert scale, ii) « small smile and smiling for more than 4 seconds OR large smile only » corresponded to a score of 2 on the Likert scale, iii) « large smile and smiling for more than 4 seconds » corresponded to a score of 3 on the Likert scale and iiii) « large smile and smiling for more than 4 seconds and Crow's feet for more than 4 seconds » corresponded to a score of 4 on the Likert scale. The global measure was determined as the sum of scores for each item (max 16 on the Likert scale). A high score on the Motor-Affective subscale of the MASS means fewer impairments of nonverbal social behaviors.

### Statistical analysis

Clinical ratings and nonverbal behavior displays were separately compared for the three groups with a non-parametric Kruskal-Wallis test. In order to compare the scores of schizophrenia patients with pro-social priming, anti-social priming, and non-social priming, non-parametric Mann-Whitney U tests were computed for each nonverbal social behavior performance. The level of significance was set to *p*<.05.

## Results

### Demographic and clinical measures

The results revealed no difference on the basic demographic and clinical information between schizophrenia groups (see Table 1).

### Nonverbal social behavior measures

**Figure 2:**
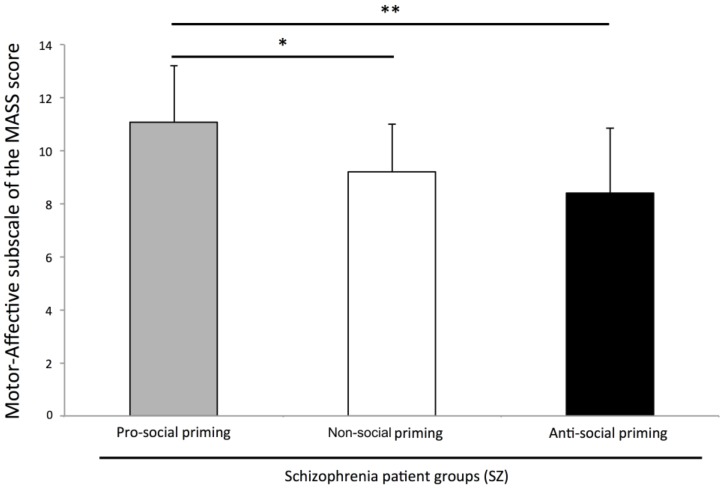
Total number of nonverbal behaviors. Schizophrenia patients (SZ) primed in pro-social condition and SZ primed in non-social and anti-social conditions were significantly different. SZ primed in non-social condition and SZ primed in anti-social condition were equivalent. Error bars represent standard deviations. * *p*<.05 and ** *p*<.001.

The Kruskal-Wallis test on the global nonverbal behaviors assessed by the Motor-Affective subscale of the MASS revealed a significant main effect for priming conditions (*H* = 9.542, *p* = .008). Contrast comparisons revealed that schizophrenia patients primed with pro-social words showed significantly more nonverbal behaviors than schizophrenia patients primed with anti-social priming words (*z* = −2.918, *p* = .004) and than schizophrenia patients primed with non-social priming words (*z* = −2.231, *p* = .026). Schizophrenia patients in non-social and anti-social priming conditions were not different (*p*>.05, see Figure 2).

**Figure 3:**
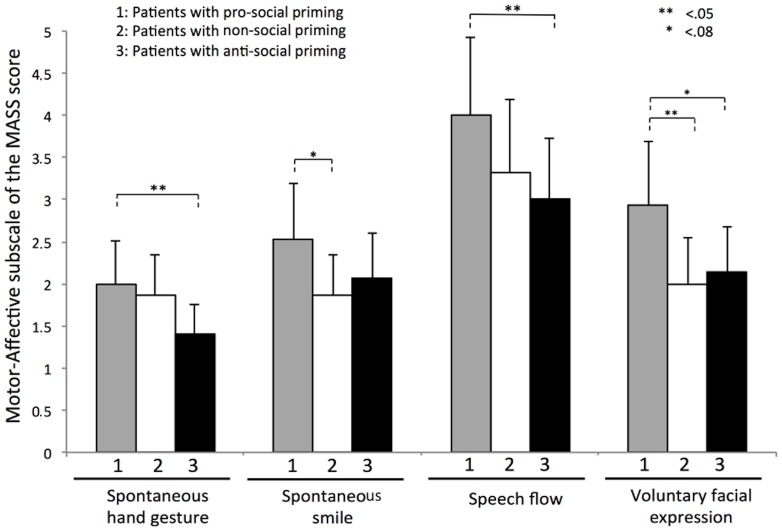
Subdimensions of nonverbal behavior. Significant differences were found between each comparison of patient groups. Error bars represent standard deviations.

Results demonstrated that schizophrenia patients primed with non-social words and patients primed with anti-social words were equivalent on all four dimensions of the Motor-Affective subscale of the MASS (spontaneous smiles, spontaneous hand gestures, speech flow and voluntary facial expression, all *p*>.05, see Figure 3). However, pro-social priming affected the different nonverbal behaviors in schizophrenia patients. Compared to anti-social priming, pro-social priming significantly increased spontaneous hand gestures score (*z* = −2.253, *p* = .024), speech flow score (*z* = −2.356, *p* = .018) and marginally enhanced voluntary facial expression score (*z* = −1.824, *p* = .068). The spontaneous smile measure was equivalent between schizophrenia groups primed with pro-social and anti-social conditions. Schizophrenia patients in pro-social priming also had a higher score on voluntary facial expression than patients with non-social priming (*z* = −2.023, *p* = .043). They also exhibited a marginally greater score on the spontaneous smiles measure (*z* = −1.814, *p* = .070). Finally, spontaneous hand gestures and speech flow dimensions were equivalent between schizophrenia patients primed with pro-social and non-social priming conditions.

## Discussion

It was shown repeatedly that goals can be activated outside of awareness through semantic priming and then nonconsciously operate to increase goal-directed behavior [30]–[36]. Concerning social priming, the goal-activation theory of Bargh et al. (2001) predicts that social priming activates an affiliation goal representation, which then automatically leads to the pursuit of that goal. In their initial study, Bargh et al. (2001) showed that nonconscious social goal activation promoted not only goal-directed action but also increased and maintained the motivation over time to achieve the social goal behavior [32]. These results have been replicated in numerous studies [2]. Surprisingly, although nonverbal social behaviors are known to be strongly impaired in schizophrenia [14]–[17], no study has tested whether social priming might affect such deficits.

The main goal of the present study was to investigate the effect of social priming on nonverbal social behaviors in schizophrenia. To this aim, we used the Scrambled-Sentence Task, inspired of Leighton et al. (2008) but initially developed by Srull and Wyer [37], to socially prime schizophrenia patients according to three priming group conditions (i.e., pro-social, anti-social and non-social conditions). Nonverbal social behaviors in a subsequent interaction were then measured. As hypothesized, schizophrenia patients in the pro-social priming condition exhibited significantly more nonverbal behaviors than schizophrenia patients primed with non-social and anti-social conditions and these two groups (non-social and anti-social conditions) were not statistically different. These results are in line with the literature [29], [38] and confirm that schizophrenia patients are sensitive to the social priming effect [13]. For the first time, results indicate that the production of nonverbal behaviors during social interaction, repeatedly shown as impaired in schizophrenia [14]–[17], can be significantly promoted using pro-social priming. More specifically, our results indicate that, in schizophrenia participants, pro-social priming significantly increased several dimensions (i.e. spontaneous hand gestures, speech flow and voluntary facial expression) of the Motor-Affective subscale of the MASS compared to the anti-social priming condition. In addition, we found that pro-social priming increased significantly voluntary facial expressions and the number of spontaneous smiles compared to the non-social condition. Importantly, equivalence of demographical and psychological variables (i.e., depression, anxiety level, symptomatological level and social functioning) between our groups suggests that our results are not due to these potential confounding variables.

A large body of evidence demonstrated that nonverbal behaviors play a key role in interpersonal human communication [39]. In a recent study, we found that fewer nonverbal behaviors during social interaction as measured by the MASS were positively correlated with a poor social functioning in schizophrenia [6]. Thus, social goal activation during therapeutic groups, such as social skills groups, could increase and help maintaining the acquisition of new social competencies by the patients. Mainly, the repetition of social priming task in schizophrenia may be used to automate this social goal activation during social interactions, and increase the achievement of social behaviors and thus, promote better social functioning in schizophrenia. Therefore, we think that this study shows that an experimental task such as a social priming task could have important clinical implications for patients. Although additional research is needed, our results suggest that social priming could be a powerful add-on clinical care tool in schizophrenia and probably in other mental disorders with severe social impairments such as social phobia or autism.

The present study has some limitations. First, the sample size for the groups was small, which may have influenced the representativeness of the samples as well as the power of the tests. Future research should confirm our findings using larger samples. A second limitation is that the Motor-Affective subscale of the MASS is limited by its item quantification on a Likert scale of 1–4, which limits the tool's sensitivity, and by the assessment of voluntary facial expression, which is quantified by only one item. A more sensitive tool could enable more accurate assessment of the global nonverbal social behaviors. A third limitation is that the second experimenter was not blind to the purpose of the study. Nevertheless it must be noted that as our study was an exploratory study without initial hypothesis that could have biased our results.

## Conclusion

Schizophrenia patients have been characterized as having a reduced number of nonverbal gestures during social interactions. Nonverbal gesture reductions have been linked to an important reduction in social functioning. Specifically targeting nonverbal behavioral deficits in this pathology seems to be of great importance to promote better quality of life of patients. Our study reveals that a social priming task can help promote nonverbal expressive behaviors during social interactions in schizophrenia patients. Social priming tasks may be useful to activate social goal during therapeutic groups activities, such as social skills training, and help facilitate the acquisition of new social competencies in patients.
